# Vascular access placement in patients with chronic kidney disease Stages 4 and 5 attending an inner city nephrology clinic: a cohort study and survey of providers

**DOI:** 10.1186/s12882-016-0431-3

**Published:** 2017-01-17

**Authors:** Narender Goel, Caroline Kwon, Teena P. Zachariah, Michael Broker, Vaughn W. Folkert, Carolyn Bauer, Michal L. Melamed

**Affiliations:** 1 Division of Nephrology, Montefiore Medical Center/Albert Einstein College of Medicine, 1300 Morris Park Avenue, Ullmann 615, Bronx, 10461 NY USA; 2 Department of Internal Medicine, Montefiore Medical Center/Albert Einstein College of Medicine, 1300 Morris Park Avenue, Ullmann 615, Bronx, 10461 NY USA; 3 Department of Epidemiology and Population Health, Montefiore Medical Center/Albert Einstein College of Medicine, 1300 Morris Park Avenue, Ullmann 615, Bronx, 10461 NY USA

**Keywords:** Vascular access, CKD, Acute kidney injury, Hospitalizations, Hemodialysis

## Abstract

**Background:**

The majority of incident hemodialysis (HD) patients initiate dialysis via catheters. We sought to identify factors associated with initiating hemodialysis with a functioning arterio-venous (AV) access.

**Methods:**

We conducted a retrospective chart review of all adult patients, age >18 years seeing a nephrologist with a diagnosis of CKD stage 4 or 5 during the study period between 06/01/2011 and 08/31/2013 to evaluate the placement of an AV access, initiation of dialysis and we conducted a survey of providers about the process.

**Results:**

The 221 patients (56% female) in the study had median age of 66 years (interquartile range (IQR), 57–75) and were followed for a median of 1.26 years (IQR 0.6–1.68). At study entry, 81%had CKD stage 4 and 19% had CKD stage 5. By the end of study, 48 patients had initiated dialysis. Thirty-four of the patients started dialysis with a catheter (1 failed and 10 maturing AVFs), 9 with an AVF and 5 with an AVG. During the study period, 61 total AV accesses were placed (54 AVF and 7 AVG). A higher urinary protein/ creatinine ratio and a lower eGFR were associated with AV access placement and dialysis initiation. A greater number of nephrology visits were associated with AV access creation but not dialysis initiation. Hospitalizations and hospitalizations with an episode of acute kidney injury (AKI) were strongly associated with dialysis initiation (odds ratio (OR) 13.0 (95% confidence interval (CI) 2.3 to 73.3, *p*-value = 0.004) and OR 6.6 (95% CI 1.9 to 22.8, *p*-value = 0.003)).

**Conclusions:**

More frequent nephrology clinic visits for patients with a recent hospitalization may improve rates of placement of an AV access. A hospitalization with AKI is strongly associated with the need for dialysis initiation. Nephrologists may not be referring the correct patients to get an AV access surgery.

## Background

Chronic kidney disease (CKD),including end-stage renal disease (ESRD) requiring dialysis or transplantation, affects 16.8% of adults in the United States (US) [1] and is associated with an increased risk of death from cardiovascular disease [2]. Hemodialysis (HD) is the most common form of renal replacement therapy in the US, and thus, particular attention must be given to the placement of a vascular access. Among all available vascular access options, an arterio-venous fistula (AVF) is preferred owing to its longevity and the fewest associated complications. An arterio-venous graft (AVG) is usually placed when a surgeon is unable to place an AVF. Catheters are the least preferred as their use has been shown to be a risk factor for bacteremia and septicemia which correlates with an increased risk of myocardial infarction, stroke, peripheral vascular disease and death [3].

In order to increase the prevalence of AVFs in ESRD patients, the National Kidney Foundation launched the Fistula First Breakthrough Initiative (FFBI) in 2003, which increased AVF use in prevalent ESRD patients from 32% in 2003 to 56% in June 2010. During the same period AVG use has decreased from 40% to 20%, however 82% of patients still initiated HD via a catheter [4].

According to the United States Renal Data System (USRDS), 80% of patients started HD via a catheter in 2013 [5]. Previous studies have shown pre-dialysis education, pre-dialysis nephrology care, more frequent clinical encounters and the presence of insurance as some of the factors associated with the use of an AV access at initiation of dialysis [6–9]. Whether or not a patient has seen a nephrologist prior to starting dialysis may be subject to error depending on the data source [10]. Earlier studies have also shown that only 50% of patients start dialysis within a year of obtaining an AV access, suggesting that nephrologists may not be recognizing which patients will need dialysis [11]. A study of ESRD in the elderly found that among patients with CKD, those who developed acute kidney injury (AKI) had a hazard ratio of developing ESRD almost 5 times the hazard ratio of those who didn’t develop AKI [12]. Thus, potentially one of the biggest risk factors for progression may be difficult to predict.

As there is limited information available about patients with late stage CKD and their specific barriers to AV access placement, we studied all adult patients with CKD stage 4 or 5, to evaluate the timeliness of AV access placement and identify barriers to access placement and factors associated with initiation of dialysis with or without an AV access. We also surveyed nephrologists at our institution to assess their perceptions of the access placement process. We hypothesized that patients seen by a nephrology fellow along with a faculty member would be more likely to have an AV access placed.

## Methods

### Patients

We conducted a retrospective chart review of all adult patients, age >18 years seeing a nephrologist with new CKD stage 4 or 5 during the study period between June 1, 2011 and August 31, 2012. Patients were followed via chart review until August 31, 2013. The patients (*n* = 31) who had prior nephrologist follow-up for CKD stage 2 or 3 but were seen during the study period for the first time with a diagnosis of CKD stage 4 or 5 were also included and their day of nephrology visit with CKD stage 4 or 5 diagnosis was considered as the initial study visit. Patients referred to the nephrology clinic at Montefiore Medical Center (MMC) are managed either by faculty nephrologists independently or in conjunction with nephrology fellows. CKD stage 4 was defined as an estimated glomerular filtration rate (eGFR) between 15–29 ml/min/1.73 m^2^ and CKD stage 5 was defined as an eGFR <15 ml/min/1.73 m^2^ at study entry. GFR was estimated using the Modification of Diet in Renal Disease (MDRD) formula by the hospital’s laboratory using creatinine measurements that are IDMS-traceable [13].

**Fig. 1:**
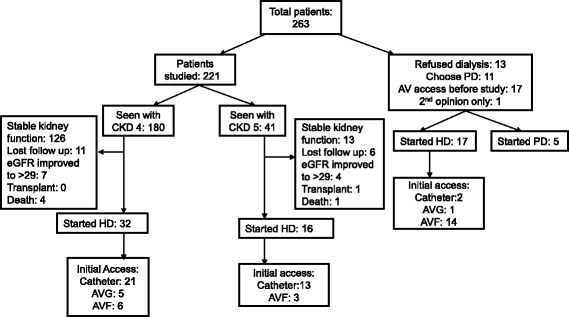
Study Flow Diagram

Patients were excluded from study (Fig. 1) if they refused dialysis (*n* = 13), decided on peritoneal dialysis (*n* = 11) or had an AV access placed before the study period (*n* = 17). These 17 patients had a previous AV access placed due to being post-transplant, transfers from other centers or post-dialysis requiring AKI with an AV access already placed. One additional patient came for a second opinion, did not return to clinic and was not included in this study. Patients were also excluded if they were seeing a nephrologist (at Montefiore) for CKD stage 4 or 5 prior to June 1, 2011.

### Measurements

Data were extracted using the Clinical Looking Glass (CLG) system (Emerging Health Information Technology, Yonkers, New York), an interactive software application developed at MMC that integrates clinical and administrative datasets [14]. We used CLG to extract demographic data, insurance status, race/ethnicity, body mass index, smoking history, primary language (English, Spanish), co-morbidities as coded by ICD-9 codes (presence or absence of diabetes mellitus, hypertension, congestive heart failure, and peripheral vascular disease) and laboratory data including hemoglobin, serum albumin and creatinine which were measured within 90 days prior to or 15 days after the initial nephrology visit for incident stage 4 and 5 CKD. When multiple values were available, the value closest to the clinic visit was used for analysis.

Electronic medical records were reviewed to get information regarding the number of nephrology visits, eGFR at the time of the visits, CKD etiology, date and eGFR at time of referrals, details of AV access surgery, or if the patient was not referred for access surgery, the reasons for non-referral. We collected data on the number of AV access placed and on the initial vascular access in patients starting HD. We also collected data on hospitalizations and about whether the patients experienced an AKI episode (defined as a creatinine rise >0.3 mg/dL) during the hospitalization. A patient was considered lost to follow up if the patient was not seen in any Montefiore clinic in last 6 months of the study period. Data on death was obtained from hospital records and through linkage to the Social Security Administration (SSA) mortality registry. This linkage occurs in a continuous fashion by CLG. Data on dialysis initiation was obtained by 1) review of electronic medical records and 2) search for billings codes for ESRD (585.6) or hemodialysis (39.95) or renal dialysis status (V45.11). Data collection ceased at time of death or dialysis whichever occurred first or at the time the patient was lost to follow-up.

### Nephrologists survey

We also conducted a web-based anonymous survey of all of the nephrology faculty members (all ABIM certified in Nephrology) and fellows (PGY 4 and 5) using REDCap software [Einstein-Montefiore Institute for Clinical and Translational Research grant support *(UL1 RR025750*)]. Questions and responses in the survey included:

1. In your opinion, what is the main limiting factor in referring patients with CKD stage 4 and 5 to a vascular surgeon?
  Possible answers:
  Patients’ refusal;
  patients’ non-compliance;
  patients not decided about modality of dialysis;
  nephrologists;
  insurance status;
  co-morbidities.
2. In your opinion, what is the main limiting factor in obtaining timely vascular access?
  Possible answers:
  Nephrologists;
  vascular surgeon;
  hospital system and appointments;
  patients;
  I am not sure.

For analysis we grouped patients’ refusal and non-compliance as patient factors, nephrologists as nephrologist factor, hospital system and appointment and insurance status as health care system factor, vascular surgeon as vascular surgeon factor. From the chart review, reasons for not getting an AV access placed were categorized as nephrologist reasons, if there was no referral to vascular surgery noted in the chart; patient reasons, if patient refused to undergo AV access surgery or if there was a referral but patient did not make the appointment; hospital system and appointments if patients were waiting for their appointment to be evaluated by vascular surgeon or to undergo AV access surgery or unable to undergo surgery due to lack of insurance.

### Statistical analysis

We first compared the characteristics of patients seen by faculty alone versus with nephrology fellows using the Mann Whitney test and chi-square tests as appropriate. The primary outcome was AV access placement. We assessed the associations of key variables with vascular surgery referral, AV access placement and initiation of dialysis separately using multivariable logistic regression. Variables of interest included care by a renal fellow, the number of nephrology visits, whether or not the patient was hospitalized and whether or not the patient had an AKI episode during the hospitalization. Due to the small number of events, we used the variables of interest separately in models adjusted for possible confounders including age, sex, race/ethnicity, diabetes mellitus, log protein/creatinine ratio and baseline eGFR.

We created a categorical variable classifying patients into 4 possible outcomes: 1) no access placed/ no dialysis initiated, 2) access placed/ no dialysis initiated, 3) no access placed, dialysis initiated and 4) access placed/dialysis initiated. We then evaluated whether there were any factors that differentiated these groups using chi-square tests or ANOVA as appropriate. We then compared the reasons obtained from the nephrologist survey to the chart review data using Chi Square tests. All statistical analysis was done using STATA version 10.2, Texas, US. All mean values were reported with one standard deviation. Two-sided *p*-value <0.05 was considered statistically significant. The study protocol was approved by the institutional review board of the Albert Einstein College of Medicine. The need for informed consent was waived due to the retrospective nature of study involving chart review only. The study was conducted in adherence with the Declaration of Helsinki.

## Results

The 221 patients (56% female) in the study had mean (± standard deviation) age of 64.8 ± 13.6 years and were followed for a median of 1.26 years (IQR 0.6–1.68 years). Out of the 221 patients, 81% had CKD stage 4 and 19% had CKD stage 5 at study entry. Fourteen percent of patients (*n* = 31) had prior follow-up with CKD stage 2 or 3 but were seen during the study period for the first time with CKD stage 4 (*n* = 30) or stage 5 (*n* = 1). The mean eGFR at study initiation was 20.8 ± 6.4 ml/min/1.73 m^2^. As expected, diabetes mellitus (30.8%) and hypertension (25.8%) were the predominant etiologies of CKD. Glomerular diseases, acute kidney injury and multifactorial causes were identified in 4.1%, 4.5% and 4.9% of patients, respectively.

Patients seen by faculty members independently as compared to patients seen in conjunction with fellows were significantly older, predominantly female and less likely to have Medicaid and more likely to have Medicare. The mean hemoglobin and albumin was significantly higher among faculty patients as compared to fellows’ patients, whereas the mean serum creatinine and urinary protein/creatinine ratios were significantly higher among the fellows’ patients (Table 1).

**Table 1:** Baseline characteristics of study patients. Data presented as either number (percent) or mean (standard deviation) as appropriate

	Total (221)	Faculty (141)	Fellow (80)	*p*-value
Age [years]	64.8 (13.6)	67.2 (12.9)	60.6 (13.7)	<0.001
Female (%)	124 (56)	91 (64.5)	33 (41.2)	0.001
Mean BMI [Kg/m^2^]	30.4 (7.0)	30.7 (7.1)	29.7 (6.9)	0.14
Co-morbidities				
Hypertension (%)	206 (93.2)	130 (92.2)	76 (95)	0.58
Diabetes Mellitus (%)	146 (66)	93 (65.9)	53 (66.3)	0.9
Congestive Heart Failure (%)	96 (43.4)	58 (41.1)	38 (47.5)	0.39
Peripheral Vascular Disease (%)	33 (14.9)	23 (16.3)	10 (12.5)	0.55
Race/ Ethnicity				0.06
White (%)	17 (7.7)	14 (9.9)	3 (3.7)	
African-American (%)	68 (30.8)	49 (34.7)	19 (23.7)	
Hispanic	107 (48.4)	63 (44.7)	44 (55)	
Other	29 (13.1)	15 (10.6)	14 (17.5)	
Primary Language				
English (%)	164 (74.2)	108 (76.5)	56 (70)	0.3
Spanish (%)	51 (23.2)	30 (21.3)	21 (26.2)	0.4
Insurance				
Medicaid (%)	77 (34.8)	33 (23.4)	44 (55)	<0.001
Medicare (%)	70 (31.8)	54 (38.3)	16 (20)	0.006
Never smoker (%)	118 (53.4)	76 (53.9)	42 (52.5)	0.8
Hemoglobin, mean (SD) [gm/dL]	10.7 (1.8)	10.9 (1.8)	10.3 (1.8)	0.04
Albumin, mean [gm/dL]	3.8 (0.6)	3.96 (0.6)	3.53 (0.7)	<0.001
Creatinine, mean [mg/dL]	2.88 (1.2)	2.7 (1.2)	3.18 (1.2)	0.005
Renal Clinic visit, mean	5.4 (4.1)	5.3 (4.2)	5.5 (4.1)	0.8
eGFR [ml/min/1.73 m^2^] at the study entry, median (IQR)	20.8 (6.4)	21.3 (6.2)	19.8 (6.5)	0.07
Urine albumin/creatinine ratio	0.78 (0.18, 3.73)	0.51 (0.13, 2.08)	2.64 (0.44, 5.31)	<0.001
Follow up (years), median (IQR)	1.26 (0.6–1.68)	1.3(0.75–1.69)	1.2 (0.4–1.6)	0.1
Any hospitalization	155 (70.1)	89 (63.1)	66 (82.5)	0.002
Any hospitalization with acute kidney injury episode	136 (61.5)	74 (52.5)	62 (77.5)	<0.001

**Fig. 2:**
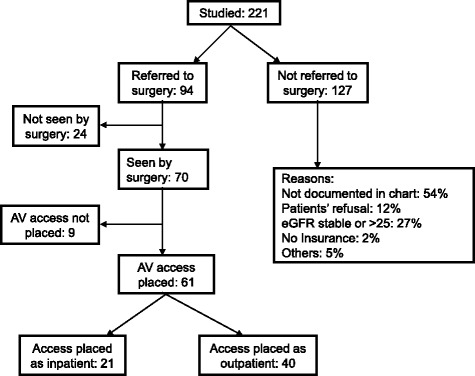
Flow Diagram showing vascular surgery referral and AV access placement

A total of 94 patients (42.5%) were referred to vascular surgery with a mean eGFR at the time of referral of 16.3 ± 5.5 ml/min/1.73 m^2^. Access surgery was done in 61 (27.6%) patients (55 AVF and 6 AVG) with mean eGFR of 14.3 ± 6.2 ml/min/1.73 m^2^ (Figs. 1 and 2). The median time of referral to the surgeon from the initial nephrology study visit was 28 days (IQR, 0–133) while the median time to see the surgeon from the time of referral was 52 days (IQR, 27–106). The median time to surgery after an appointment with the surgeon was 30 days (IQR, 15–85).

Factors associated with vascular surgery referral were African-American race, a higher number of nephrology clinic visits, lower eGFR and higher urine protein/ creatinine ratio (Table 2). Being seen with a renal fellow was not shown to be associated with vascular surgery referral. The only factors shown to be associated with the placement of an AV access were a higher number of nephrology clinic visits, a higher protein/creatinine ratio and lower eGFR at entry to the study. Factors associated with initiation of dialysis were a higher urine protein/creatinine ratio, a lower baseline eGFR and having a hospitalization during follow-up or a hospitalization with an AKI episode during follow-up. Of the 48 patients who started dialysis, 44 of them had a hospitalization with an AKI episode, compared to 4 such hospitalizations in 173 patients who did not start dialysis (*p*-value <0.001 for comparison). The predominant reasons for not undergoing an access surgery (*n* = 160) were as follows; 43% of patients were not referred for unknown reasons, 20% of patients had stable eGFR or eGFR >25 ml/min/1.73 m^2^, 10% of patients refused and 7% of patients missed their appointment.

**Table 2:** Odds ratio of vascular surgery referral and AV access placement

	Vascular surgery referral (*n* = 94)			AV access placement (*n* = 61)			Initiated Dialysis (*n* = 48)		
	OR^a^	95% CI	*p*-value	OR^a^	95% CI	*p*-value	OR	95% CI	*p*-value
Age, per year	0.99	0.96 to 1.02	0.33	0.97	0.94 to 1.00	0.06	0.98	0.95 to 1.01	0.27
African-American Race (compared to white)	4.65	1.00 to 21.6	0.05	1.10	0.27 to 4.46	0.89	0.72	0.15 to 3.43	0.68
Hispanic Ethnicity (compared to non-Hispanic white)	2.81	0.64 to 12.44	0.17	0.70	0.18 to 2.76	0.61	0.51	0.11 to 2.31	0.38
Diabetes Mellitus	1.29	0.58 to 2.88	0.53	0.91	0.40 to 2.06	0.82	1.76	0.66 to 4.71	0.26
Log urine protein/ creatinine ratio	1.45	1.13 to 1.86	0.003	1.36	1.05 to 1.75	0.02	1.72	1.28 to 2.32	<0.001
eGFR at the study entry	0.87	0.82 to 0.93	<0.001	0.89	0.83 to 0.94	<0.001	0.90	0.84 to 0.97	0.003
Patient seen with renal fellow	1.45	0.67 to 3.13	0.34	1.10	0.25 to 1.49	0.82	1.35	0.56 to 3.27	0.50
Number of nephrology visits	1.27	1.12 to1.45	<0.001	1.13	1.01 to1.25	0.03	1.02	0.92 to 1.14	0.68
Hospitalization during follow-up	0.97	0.41 to 2.29	0.94	2.46	0.94 to 6.4	0.07	13.0	2.3 to 73.3	0.004
AKI during hospitalization	0.78	0.35 to 1.72	0.53	1.84	0.79 to 4.28	0.26	6.6	1.89 to 22.8	0.003

^a^All models adjusted for age, sex, race/ethnicity, diabetes mellitus, log urinary albumin/creatinine ratio and baseline eGFR. Renal fellow visit, number of renal visits, number of hospitalization, and the presence of AKI during a hospitalization put in individually with the above adjusters. (abbreviations: OR-odds ratio; CI-confidence interval)

Patients who started HD but were not referred for vascular surgery were more likely to have diabetes mellitus, lower hemoglobin, albumin and eGFR levels and higher urinary protein/creatinine ratios compared to patients who did not start HD and not referred. All the patients who started HD and were not referred for vascular surgery had had a hospitalization with an AKI episode compared to only 52% of patients who did not start dialysis and were not referred (*p* <0.001) (Table 3).

**Table 3:** Characteristics of patients who started dialysis compared to patients who had an AV access placed during the follow-up period

Characteristic	No HD, No referral (*n* = 114)	Started HD, no referral (*n* = 13)	No HD, Referred for AV access (*n* = 59)	Started HD and Referred for AV access (*n* = 35)	*p*-value
Age, mean (SD)	68.0 (13.3)	60.5 (15.5)	63.3 (13.2)	58.9 (11.7)	0.002
Female (%)	72 (63)	7 (53)	26 (44)	19 (54)	0.12
Mean BMI [Kg/m^2^]	31.0 (7.4)	28.7 (9.1)	29.5 (6.0)	30.4 (6.4)	0.47
Co-morbidities					
Hypertension (%)	104 (91)	12 (92)	57 (97)	33 (94)	0.60
Diabetes Mellitus (%)	68 (60)	10 (77)	38 (64)	30 (86)	0.03
Congestive Heart Failure (%)	45 (39)	9 (69)	24 (41)	18 (51)	0.15
Peripheral Vascular Disease (%)	16 (14)	3 (23)	9 (15)	5 (14)	0.86
Race/ Ethnicity					0.15
White N (%)	11 (10)	3 (23)	2 (3)	1 (3)	
African American(%)^a^	33 (29)	6 (46)	19 (32)	10 (29)	
Hispanic N (%)	52 (45)	4 (31)	30 (51)	21 (60)	
Other N (%)	18 (16)	0	8 (14)	3 (8)	
Primary Language					0.74
English (%)^a^	82 (72)	12 (92)	44 (75)	26 (74)	
Spanish (%)^a^	29 (25)	1 (8)	12 (20)	9 (26)	
Never smoker (%)	67 (59)	4 (31)	30 (50)	17 (49)	0.45
Hemoglobin, mean (SD) [gm/dL]	11.1 (1.8)	9.2 (2.1)	10.5 (1.7)	10.5 (1.9)	0.001
Albumin, mean [gm/dL]	4.02 (0.55)	3.36 (0.68)	3.77 (0.63)	3.45 (0.59)	<0.001
Creatinine, mean [mg/dL]	2.39 (0.80)	3.5 (1.93)	3.04 (1.00)	3.95 (1.46)	<0.001
Hospitalization, N (%)	70 (61)	13 (100)	39 (66)	33 (94)	<0.001
Renal Clinic visit, median (IQR)	4 (2 to 7)	2 (1 to 4)	6 (3 to 11)	3 (6 to 9)	<0.001
eGFR [ml/min/1.73 m^2^] at the study entry, median (IQR)	26 (21 to 28)	20 (16 to 27)	19 (15 to 25)	17 (12 to 20)	<0.001
Hospitalization with AKI episode, N (%)	59 (52)	13 (100)	33 (56)	31 (89)	<0.001
Urine albumin/creatinine ratio	0.27 (0.1 to 0.82)	3.83 (1.87 to 5.15)	1.48 (0.61 to 5.07)	4.35 (2.38 to 7.87)	0.22
Follow up (years), median (IQR)	1.2 (0.6–1.7)	0.6 (0.3–1.0)	1.3 (1.1–1.7)	1.3 (0.5–1.5)	

^a^Total may not add up to 100% due to missing values

By the end of study, 48 patients had started HD with mean eGFR of 9.0 ± 4.9 ml/min/1.73 m^2^ (Fig. 1). Of all the patients started on HD, 30 patients (62.5%) saw a nephrologist for less than a year and 17 patients (35.4%) had seen the nephrologist for <6 months. The mean time from the study visit to hemodialysis was similar in patients with initial nephrology visit with CKD stage 5 and CKD stage 4 (0.68 ± 0.5 years and 0.83 ± 0.5 years, *p* = 0.4).

In the survey of 17 out of 30 nephrologists at our institution (57% response rate), patient related factors were thought to be the major limiting factor in vascular surgery referral (88%) and AV access placement (41%). Chart review revealed nephrologists to be the major limiting factors in 51% and 44% cases, respectively (Table 4).

**Table 4:** Reasons for non-placement of vascular access

Limiting Factors	Vascular Surgery Referral			AV Access placement		
	Nephrologist Survey	Observed by chart Review	*p*-value	Nephrologist Survey	Observed by chart review	*p*-value
Patients	88^a^%	15%	<0.001	41%	18%	0.01
Nephrologists	6%	51%	<0.001	6%	44%	<0.001
Health system problems^b^	6%	2%	0.19	41%	11%	<0.001
Vascular surgeon	NA	NA	NA	0%	0%	NA
Stable GFR^c^	NA	27%	NA	NA	20%	NA
Others	NA	5%	NA	12^d^%	8%	0.5

^a^Patient refusal (47%), patient non-compliance (29.4%) and patient not decided about modality of dialysis (11.8%); ^b^Health system problems include insurance problems and hospital system and appointment problems including time delay in waiting for surgery or appointment. ^c^It was not known to be a barrier at the time of survey hence was not included in survey; ^d^Actual answer: “I am not sure”; Abbreviation: NA-Not applicable

## Discussion

Our study shows that in patients with diagnosis of CKD stage 4 or 5 at our institution, the majority of patients initiate hemodialysis via catheters. This is especially true in those who saw a nephrologist for less than a year (86.6%) and those with CKD stage 5 at referral (87.5%). Although a much higher proportion of patients with a first nephrology visit with CKD 5 (41%) required dialysis during the study period as compared to CKD 4 (18.3%), the mean time to dialysis initiation was comparable in both groups. Hence, it is important to promptly refer patients for AV access placement who are at high risk of progression to ESRD needing hemodialysis. It is, however, hard for nephrologists to predict which patients will require dialysis [11]. Tangri et al. and Landray et al. have proposed predictive models which may be used to identify patients for ESRD progression [15, 16]. In our late stage CKD population, hospitalizations and hospitalizations with an AKI episode were strongly associated with the need for dialysis suggesting that nephrologists need to be vigilant with these patients and follow them frequently in clinic.

During the study period, 57% of patients were not referred to vascular surgery and 72% of all patients didn’t undergo AV access placement. During this short follow up with a median number of 4 (IQR 2–8) [mean 5.4 ± 4.1] nephrology visits, it appears difficult to achieve successful placement of an AV access. Hence, late referrals to nephrologists, limited follow-up time, and the nephrologists’ lack of prompt referrals to surgery all together resulted in the predominant use of catheters as an initial vascular access. In a study of vascular access placement, patients with a late nephrology referral had only an 8% likelihood of having AV access creation compared to 39% of patients with early referrals [17]. Even in patients who had an AVF, many were not mature by the time the patients started dialysis and the patients still required a catheter (11 patients started with a catheter but had an AVF which was either maturing (10) or failed (1)). One factor associated with placement of a vascular access was frequent nephrology visits, suggesting that late stage CKD patients may require more frequent clinical visits. We also found that nephrologists perceive patients as the major limiting factor to vascular access placement, however, our chart review showed the nephrologist as a potential barrier. Our results suggest the need to educate nephrologists on the importance of being a leader in obtaining AV access.

The drive to avoid catheters is due to increased risk of bacteremia, metastatic infections, thrombosis, venous stenosis, need of frequent interventions to maintain their function, increased cost, anemia, cardiovascular events, hospitalizations, and mortality [18]. According to a recent study, creating early AV access may even have an added benefit of decelerating the decline of eGFR in patients with CKD, delaying the onset of ESRD [19]. While it is not clear what the physiologic mechanism of this deceleration is, or if even the deceleration was simply due to more attentive nephrology care for patients that underwent an AV access procedure, the authors of that study speculated that remote ischemic preconditioning caused by the creation of vascular access, may stimulate the release of humoral factors such as adenosine, erythropoietin and nitric oxide that are renal protective [19]. In order to reduce catheter use, planning for an early placement of an AVF is crucial to its success due to the need for maturation of veins and to account for possible failure of maturation. KDOQI guidelines suggested that patients should be referred to vascular surgeon for creation of an AVF when the eGFR is <25 ml/min/1.73 m^2^ [20]. As per DOPPS II data, in countries such as Australia/New Zealand, Germany, and Japan 50–72% of incident ESRD patients started HD with an AVF compared to 16% in the US [21]. This study also showed that countries such as Germany and Japan had much faster referral times to see a surgeon compared to the US [21]. Interestingly, an AVF may not always be the best initial access placed in some patients as previously suggested by Kurella Tamura et al. and DeSilva et al. who suggest that an AVG may be better for older patients [22, 23]. This is also suggested by our data as 11 patients started dialysis with a catheter and a “maturing AVF”.

A study of 319 patients by Lopez-Vargas et al [8] recognized that late referral by a nephrologist is an important factor similar to our study. Similarly, the study by Stehman-Breen et al [24] suggested that fewer nephrology visits (<5) prior to initiation to dialysis was associated with decreased odds of permanent vascular access at the initiation of dialysis. Both of these findings are further consolidated by our observation in this study.

Our study provides further insight in to potential barriers involved once a patient is seen by a nephrologist. FFBI suggests educating and providing feedback to nephrologists and vascular surgeons about their center specific data on vascular access placement and the catheter rate in incident patients [25]. Documentation of discussion about renal replacement therapy and plan for AV access placement should be routine [25]. Other suggestions include patient education and developing a hospital system whereby patients with CKD could be identified early and referred timely to nephrologist and/or vascular surgeon [25]. It is also important to identify patients with advanced kidney disease who are at risk of rapid progression to ESRD and need prompt referral to surgeons. From our study, a hospitalization with an AKI episode is one possible way to identify high risk patients. Models predicting patients’ progression to ESRD [15, 16] may be helpful. Appointing a dedicated vascular access nurse has shown to increase the proportions of incident ESRD patients starting HD with AVF from 56% to 75% [26]. Since the implementation of FFBI, which was also later publicized as “catheter last” and “not fistula only”, various quality improvement projects have been successful in improving AV access placement in ESRD patients [27–29].

There are limitations to our study. The chart review was performed retrospectively and thus we didn’t have information on reasons for not referring to a surgeon when not documented in chart. Our definition of an AKI episode, a creatinine rise of 0.3 mg/dL, represents a small decrement in kidney function in patients with advanced CKD which may introduce misclassification of AKI. The survey of nephrologists had a 57% response rate and is subject, like all surveys, to recall bias. We also lacked information on patients who may have initiated HD at other institutions or at an outpatient HD unit and were never seen at our institution thereafter. However, Montefiore Medical Center is the largest provider of care to patients in the Bronx and provides care to over 500,000 of the 1.4 million individuals living in the Bronx. Our ascertainment of deaths was limited by potential time lag from the use of SSA data. Finally, it is a single center study with small numbers. However, there are several strengths to our study. These include a detailed chart review, linkage to mortality data, and prospectively collected data on Nephrologists’ attitudes about vascular access placement.

## Conclusions

In conclusion, despite the knowledge that starting HD with an AV access is optimal for patient care and survival, we found that late referral to vascular surgeons continue to be a major obstacle in obtaining timely AV access. Earlier referral to nephrologists and surgeons may improve rates of placement of vascular access. In addition, nephrologists may not be referring the correct patients to get an AV access surgery. A hospitalization with AKI is strongly associated with the need for dialysis in patients with CKD stages 4 and 5. However, it is hard for nephrologists to predict AKI. More frequent nephrology clinic visits for patients with a recent hospitalization may improve rates of placement of an AV access, as the number of nephrology visits was the only easily modifiable factor associated with increased likelihood of an AV access placement.

## Abbreviations

ABIM: American board of internal medicine
AKI: Acute kidney injury
ANOVA: Analysis of variance
AV: Arterio-venous
AVF: Arterio-venous fistula
AVG: Arterio-venous graft
CKD: Chronic kidney disease
CLG: Clinical looking glass
DOPPS: Dialysis outcomes and practice patterns study
eGFR: Estimated glomerular filtration rate
ESRD: End-stage renal disease
FFBI: Fistula first breakthrough initiative
HD: Hemodialysis
ICD: International classification of diseases
IQR: Interquartile range
KDOQI: Kidney disease outcomes quality initiative
MDRD: Modification of diet in renal disease
MMC: Montefiore medical center
OR: Odds ratio
SSA: Social security administration
USRDS: United States Renal Data System.
